# New shore bug (Hemiptera, Heteroptera, Saldidae) from the Early Cretaceous of China with phylogenetic analyses

**DOI:** 10.3897/zookeys.130.1563

**Published:** 2011-09-24

**Authors:** Weiting Zhang, Yunzhi Yao, Dong Ren

**Affiliations:** College of Life Sciences, Capital Normal University, 105 Xisanhuanbeilu, Haidian District, Beijing 100048, China

**Keywords:** Saldidae, fossil, phylogeny, Early Cretaceous, China

## Abstract

A new genus with a new species of Saldidae, *Brevrimatus pulchalifer* **gen. et sp. n.**, is described and illustrated. The fossil specimen was found from the Early Cretaceous Yixian Formation of Duolun County, Inner Mongolia, China. Phylogenetic analyses within Saldidae were performed, and the results indicate *Brevrimatus pulchalifer* **gen. et sp. n.** should be assigned to the subfamily Chiloxanthinae.

## Introduction

The Saldidae is a small family of insects belonging to Heteroptera. About 335 extant species have been described in this cosmopolitan family (Schuh and Polhemus 2009). Most saldids are littoral, inhabiting lake shores, beaches and stream banks and they are predaceous, feeding on small insects and decaying animal materials (Brooks and Kelton 1967).

Cobben (1959) proposed a classification of Saldidae, and divided Saldidae into three subfamilies: Aepophilinae, Chiloxanthinae and Saldinae. Schuh and Polhemus (1980) later considered the Aepophilinae to be of family rank based on their cladistic and phenetic analysis of the infraorder Leptopodomorpha. At present, Saldidae is divided into two subfamilies, Chiloxanthinae and Saldinae (Schuh and Slater 1995). The phylogenetic analyses concerning relationships within Saldidae (Polhemus 1977, Schuh and Polhemus 2009) present valuable information and conclusions.

To date, 6 incontrovertible fossil species in 3 generahave been reported: *Oligosaldina* Statz & Wagner, 1950 with three species, *Oligosaldina rottensis*, *Oligosaldina rhenana* and *Oligosaldina aquatilis*, found from Upper Oligocene deposits in Germany; *Propentacora froeschneri* (= *Oreokora froeschneri*) found in Miocene Latah Formation in USA (Lewis 1969); *Salda exigua* Germar & Berendt, 1856 found in Eocene Baltic amber, and *Salda littoralis* found in Recent Late Glacial clay (Jessen 1923).

However, 2 genera assigned to this group previously are not saldids. *Leptosalda chiapensis* (Cobben, 1971) from Mexico amber was assigned to the subfamily Leptosaldinae within Saldidae first, but was later transferred to Leptopodidae by Schuh and Polhemus (1980). Popov (1973) erected a subfamily Saldoniinae in Saldidae with one genus *Saldonia* and one species *Saldonia rasnitsyni* Popov, 1973, but later (Popov 1985) transferred the genus to Archegocimicidae, synonymized Saldoniinae under Archegocimicidae, and added two more species *Saldonia sibirica* Popov, 1985 and *Saldonia maculata* Popov, 1985, all from the Lower or Middle Jurassic of Transbaikalia, Russia. Archegocimicidae is similar to Saldidae, and it was assigned to the infraorder Leptopodomorpha (Popov 1985, 1989, Popov et al. 1994). Polhemus (1977) thought *Saldonia* probably should be classified into Dipsocoridae based on its wing venation. Cobben (1987) didn’t consider this genus as a member of the infraorder Leptopodomorpha, but he didn’t give detailed explanation.

In this paper, we described a new fossil shore bug, *Brevrimatus pulchalifer* gen. et sp. n., from the Yixian Formation, Baitugou, Nanyingpan Village, Sanbeigou Town, Duolun County, Inner Mongolia, China. Xing et al. (2005) and Zhang et al. (2004), respectively, based on isotope data and abundant statistical analysis of fossils data came to the consistent opinion that the age of the Yixian Formation is Early Cretaceous. And this opinion has been accepted widely (Swisher et al. 1999, Lu 2000, Zhou et al. 2003, Fürsich et al. 2007). Here we consider the age of the Yixian Formation as the Early Cretaceous (about 125 Ma).

## Material and methods

Our fossil specimen is deposited in the Key Laboratory of Insect Evolution and Environmental Changes, Capital Normal University, Beijing, China. It was examined with the LEICA MZ 12.5 dissecting microscope. The specimens were examined without alcohol and under alcohol. Photos were taken by a Nikon Digital Camera DXM1200C. Line drawings were made with Photoshop graphic software. Morphological terminology used here follows that of Schuh and Slater (1995).

The body length was measured from the apex of head to the apex of abdomen; body width, at the maximal width of body; pronotum length, along the midline; pronotum width, across the broadest part at its posterior angles; wing length, from the basal to the apex of anterior margin; wing width, at the maximal width of the wing. All measurements are in millimeters (mm).

## Systematic paleontology

**Order Hemiptera Linnaeus, 1758**

**Suborder Heteroptera Latreille, 1810**

**Infraorder Leptopodomorpha Popov, 1971**

**Family Saldidae Amyot & Serville, 1843**

**Subfamily Chiloxanthinae Cobben, 1959**

### Brevrimatus gen. n.

urn:lsid:zoobank.org:act:B57D1B53-16FE-421A-BCA6-8F3141142A84

http://species-id.net/wiki/Brevrimatus

#### Type species.

*Brevrimatus pulchalifer* sp. n.

#### Diagnosis.

Body ovate, moderate in size, macropterous. Head relatively short. Rostrum reaching to the base of hind coxae. Corium with large pale spots, medial fracture short, costal fracture of hemelytra very long, hypocostal ridge and associated secondary hypocostal ridge present on hemelytra, membrane with five closed cells. Posterior margin of female sternum VII concave along the midline. Base of ovipositor exposed.

#### Etymology.

 The generic name is a combination of the Latin prefix “*brev-*” (short) and Latin word “*rimatus*” (fracture), which indicated the genus with short medial fracture. Gender masculine.

#### Distribution.

 China.

### Brevrimatus pulchalifer sp. n.

urn:lsid:zoobank.org:act:80999FB3-E52E-459A-8A25-46CEAF6D947E

http://species-id.net/wiki/Brevrimatus_pulchalifer

Figs 1 2

#### Type material.

 Holotype, ♀, CNU-HET-ND2010334 p/c (part and counterpart).

#### Type locality and horizon.

 Baitugou, Nanyingpan Village, Sanbeigou Town, Duolun County, Inner Mongolia, China, Yixian Formation. Early Cretaceous.

#### Diagnosis.

 Head relatively short. The last segment of antennae slightly swollen. Corium with three large pale spots, medial fracture short, costal fracture of hemelytra very long; membrane with five cells, apex of innermost cell of membrane extending past apex of outermost cell. Posterior margin of female sternum VII extremely concave along the midline.

#### Description.

 Body ovate, about 2.4 times as long as wide.

Head 1.4 times as wide as long. Antennae slender, 4-segmented, first segment shortest, second segment longest, 1.47 times as long as the third segment, fourth segment slightly shorter than third segment. Eyes reniform, moderately protrusive, located at the posterolateral angles of the head. Ocelli round, raised slightly, ocelli separated by 1.3 times the width of an ocellus, ocelli closer to each other than to margins of eyes. Rostrum reaching to the hind coxae. Length of head subequal to the length of pronotum on midline.

Pronotum transverse, 3.2 times as wide as long, Anterior and posterior margins of pronotum concave, lateral margins straight, anterior and posterior angles feebly rounded. Scutellum distinctly longer than pronotum on midline, triangular, 1.3 times as wide as long. Tarsal formula: 3–3–3. Fore tibiae about 2.0 times as long as corresponding tarsi, fore tarsomere I shortest, tarsomeres II and III almost subequal in length; mid femora 1.3 times as long as tibiae, tibiae 2.3 times as long as tarsi, tarsomere I shortest, tarsomere II slightly longer than tarsomere III; hind tibiae long, almost 1.5 times as long as hind femora, and 2.3 times as long as tarsi. Fore wing macropterous, 0.6 times as long as body; corium and membrane clearly delimited; corium with embolium; medial fracture short, 0.3 times as long as fore wing; costal fracture of hemelytra very long, reaching to the middle of the corium; venation of corium weakly indicated; membrane large, with five closed cells, cells reduced gradually from the inner to the outer. Claval commissure shorter than scutellum length at median line. Hemelytra with only slight modification for mating, the embolar region slightly thickened.

Anterior margin of female sternum VII curve; posterior margin of female sternum VII extremely concave along the midline. Base of ovipositor exposed ventrally.

**Measurements (in mm).** Body length 8.00, width 3.18. Head length 0.84, width 1.24. Antennal measurements I–IV: 0.56, 1.30, 0.92, 0.85. Interocular space of ocelli 0.12. Interocular space of eyes 0.84. Pronotum length 0.78, width 2.52. Scutellum length 1.43, width 1.78. Length fore leg: tibia 1.22, tarsomeres I–III: 0.13, 0.23, 0.23; length mid leg: femur 1.91, tibia 1.57, tarsomeres I–III: 0.18, 0.27, 0.23; length hind leg: femur 2.14, tibia 3.15, tarsomeres I–III: 0.22, 0.69, 0.52. Hemelytron length 5.14, width 1.73.

**Figure 1.**
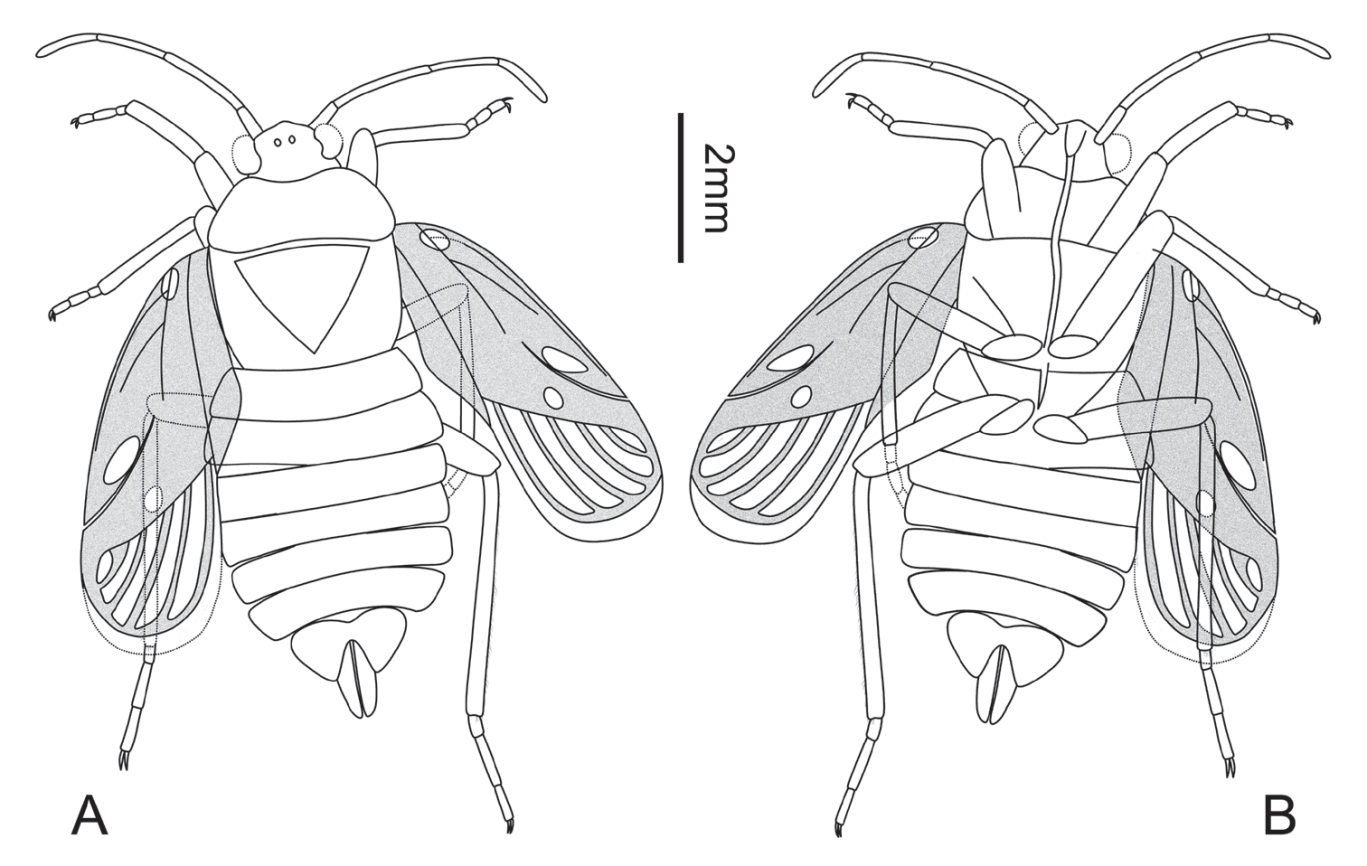
*Brevrimatus pulchalifer* gen. et sp. n., line drawings. Holotype, CNU-HET-ND2010334 p/c. **A** dorsal view **B** ventral view. Scale bar=2 mm.

**Figure 2.**
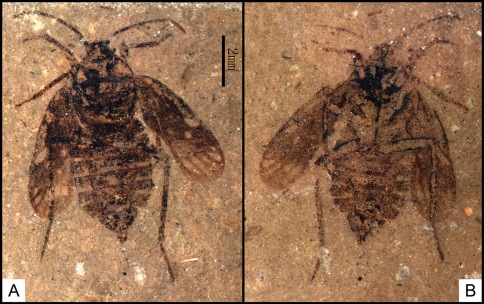
*Brevrimatus pulchalifer* gen. et sp. n., photographs. Holotype, CNU-HET-ND2010334 p/c. **A**part and **B** counterpart. Scale bar=2 mm.

#### Etymology.

 The species name is a combination of the Latin prefix “*pulch-*” (beautiful) and Latin word “*alifer*” (wing), meaning beautiful wing. Gender masculine.

## Discussion

The Leptopodomorpha consists of four extant families (Saldidae, Aepophilidae, Leptopodidae, Omaniidae) and three extinct families (Archegocimicidae, Mesolygaeidae, Palaeoleptidae). Popov et al. (1994) synonymized Mesolygaeidae to Archegocimicidae. But herein we think it is better to treat them as two separated families, because of their distinct difference in forewing. We compared our fossil with all the families in Leptopodomorpha. The body sizes of aepophilids and omaniids are less than 2mm, while the new species reaches to 8mm, much larger than aepophilids and omaniids. In Leptopodidae, rostrum at most reaches to the base of the fore coxae, while rostrum of the new species reaches to the base of the hind coxae. Besides that, anterior margin of pronotum is distinctly narrower than head in Leptopodidae, but anterior margin of pronotum of the new species is almost as wide as head. All the extinct families from Mesozoic are contemporaneous with the new fossil species. But they are different in some characters. Nine cells present in Archegocimicidae (Handlirsch 1906–1908), and the arrangement of the cells (Popov 1985) are totally different from the new species. Fore wing of Palaeoleptidae is nearly completely coriaceous except for small membrane (Poinar and Buckley 2009), which is different from the new species with large membrane. And wing venation consists of eight cells in Palaeoleptidae, which differs from the new species with five cells. The pronotum of Mesolygaeidae is divided into two parts (Zhang 1991), but in the new species no groove present on pronotum. The structure of end of abdomen is also different between the new species and mesolygaeids. So we classified our fossil into Saldidae based on the combined characters: compound eyes large and reniform, rostrum long, posterior margin of pronotum indented, hemelytra with costal fracture, medial fracture well developed and membrane with five cells.

### Phylogenetic analysis

The new genus possesses some typical Chiloxanthinae characters, such as costal fracture very long, female sternum VII truncate with mesal concavity and base of ovipositor exposed. On the other hand, it possesses short medial fracture as Saldinae. Therefore, we carried out phylogenetic analyses to determine the placement of our new genus.

For the phylogenetic analyses, we selected three extant genera from Chiloxanthinae, five extant genera from Saldinae, our new fossil genus, and an unambiguous fossil species *Oligosaldina aquatilis* as in-group. Following previous studies (Polhemus 1977, Schuh and Polhemus 1980, 2009), we chose representatives from the family Leptopodidae (*Patapius thaiensis* Cobben, 1968) and Aepophilidae (*Aepophilus bonnairei* Signoret, 1879) as our out-group taxa. The 12 taxa that we chose for these phylogenetic analyses are listed in Table 1. We carried out phylogenetic analyses respectively with the fossil taxon *Oligosaldina aquatilis* andwithout this fossil taxon.

**Table 1.** Taxa included in the phylogenetic analysis (*: only included when we carried out phylogenetic analysis with *Oligosaldina aquatilis*)

	*Family*	*Subfamily*	*Tribe*	*Species*
out-group	Leptopodidae			*Patapius thaiensis* Cobben, 1968
	Aepophilidae			*Aepophilus bonnairei* Signoret, 1879
in-group	Saldidae	Saldinae	Saldini	*Salda lugubris* (Say, 1832)
				*Teloleuca altaica* Vinokurov, 2009
			Saldoidini	*Saldula montana* Cobben, 1966
				*Calacanthia sichuanicus* Chen & Zheng, 1987
			Saldunculini	*Salduncula swezeyi* (Usinger, 1946)
		Chiloxanthinae		*Chiloxanthus pilosus* (Fallén, 1807)
				*Pentacora ligata* (Say, 1832)
				*Paralosalda innova* Polhemus & Evans, 1969
				*Oligosaldina aquatilis Statz & Wagner, 1950
				*Brevrimatus pulchalifer* gen. et sp. n.

Most character information of the extent taxa was extracted from literatures (Cobben 1959, 1969, Drake 1961, Cobben and Polhemus 1966, Polhemus and Evans 1969, Polhemus 1972, 1977, 1991, Cobben 1980, King and Fordy 1984, Chen and Zheng 1987, Vinokurov 2005, 2009, Schuh and Polhemus 2009). The descriptions for the 17 characters and character states are listed in the Appendix. All characters were treated as unordered and weighted equally. A maximum parsimony analysis of the character matrix (Table 2) edited by NDE (Nexus Data Editor) version 0.5.0 (Page 2001), was performed on NONA (Goloboff 1998), using the Multiple TBR+TBR search strategy, options set to hold 10000 trees, 1000 replications with 100 starting tree replication. The unambiguous characters were mapped by WinClada (Nixon 2000).

**Table 2.** Matrix of 17 characters and the 12 taxa used for phylogenetic analysis (*: only included when we carried out phylogenetic analysis with *Oligosaldina aquatilis*)

										1	1	1	1	1	1	1	1
Taxon/Character	1	2	3	4	5	6	7	8	9	0	1	2	3	4	5	6	7
*Patapius thaiensis*	0	0	1	0	0	0	0	1	0	–	1	0	?	0	0	0	–
*Aepophilus bonnairei*	–	2	0	0	2	–	0	0	0	–	0	0	0	0	0	0	–
*Salda lugubris*	1	?	1	1	0	0	1	1	1	0	1	0	1	1	1	0	–
*Teloleuca altaica*	1	1	1	1	0	0	1	1	1	0	1	0	1	1	1	0	–
*Saldula montana*	0	1	1	1	0	0	1	1	1	0	1	0	1	1	1	1	0
*Calacanthia sichuanicus*	1	2	1	1	1	0	1	1	1	0	1	0	1	1	1	1	0
*Salduncula swezeyi*	2	?	0	1	0	0	1	1	1	0	1	0	1	1	1	1	0
*Chiloxanthus pilosus*	2	?	0	1	0	1	2	2	1	1	2	1	0	0	1	1	1
*Pentacora ligata*	1	2	0	1	0	1	2	2	1	1	2	1	0	0	1	1	1
*Paralosalda innova*	2	2	0	1	0	0	2	2	1	1	2	1	0	0	1	1	1
*Brevrimatus pulchalifer* gen. et sp. n.	2	2	?	1	0	1	2	1	?	?	2	1	?	?	?	?	?
*Oligosaldina aquatilis	?	2	0	1	0	1	0	?	?	?	?	?	?	?	?	?	?

### Phylogenetic results

**Figure 3.**
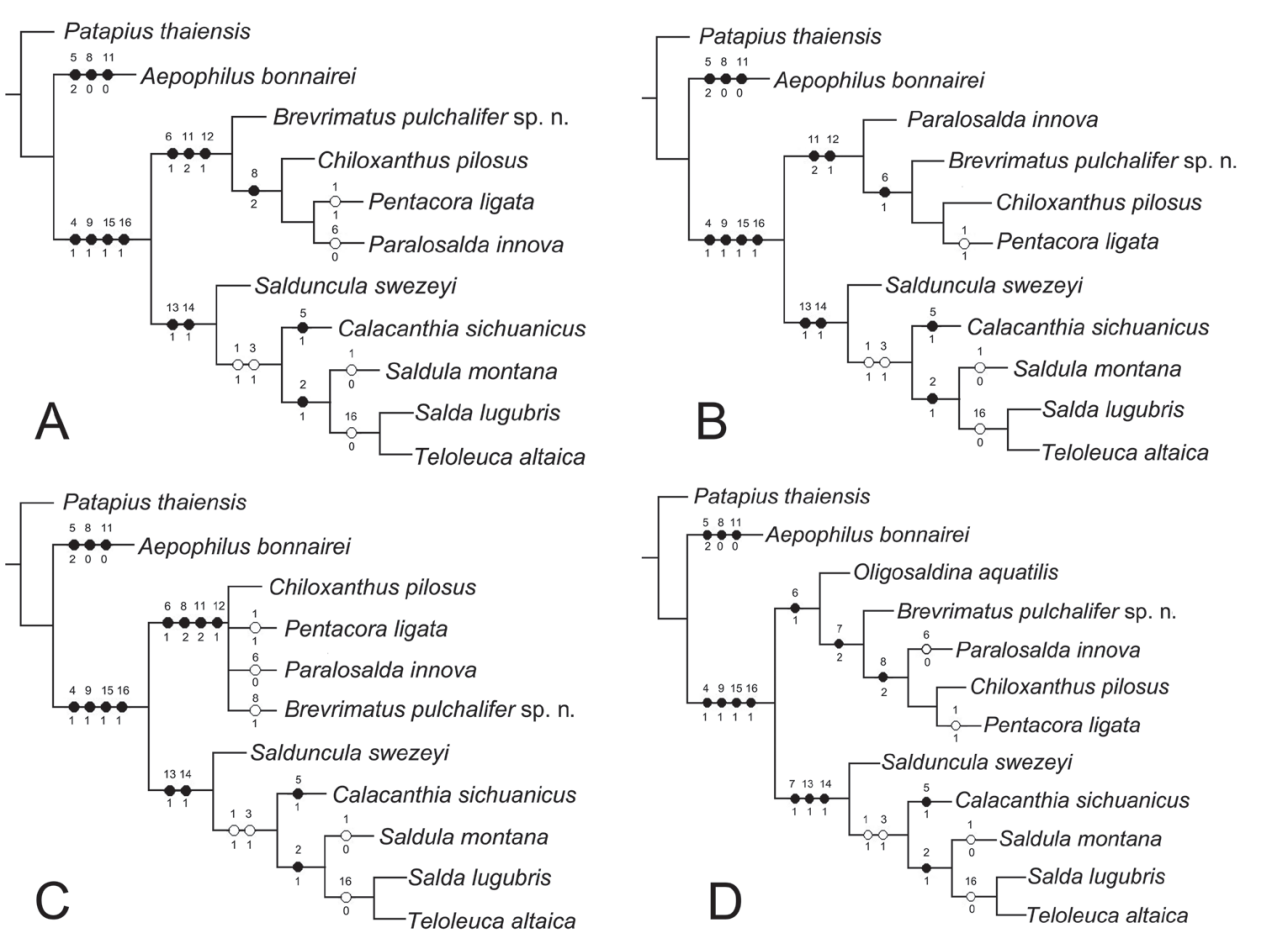
Phylogeny of Saldidae. **A, B**the most parsimonious trees based on 11 taxa and 17 characters. **C** the strict consensus tree based on 11 taxa and 17 characters **D** the most parsimonious trees based on 12 taxa and 17 characters. (●) non-homoplasious; (○) homoplasious.

For the phylogenetic analyses excluding fossil species *Oligosaldina aquatilis*, we got two equally most parsimonious trees (Fig. 3A, B), with the following main characteristics: tree length = 28, consistency index (CI) = 82, retention index (RI) = 87. The strict consensus tree is shown in Figure 3C. Phylogenetic resultsindicate Saldidae is a monophyletic group, which is supported by four synapomorphies: posterior pronotal margin indented distinctly (Character 4:1); eversible glands present posterolaterally between sterna VI and VII (Character 9:1); eggs with aeropyles (Character 15:1); larval organ present (Character 16:1). Some synapomorphic characters, such as apicolateral sclerotized structures of penis present (Character 13:1) and filum gonopori coiled one to four times, like a watch-spring (Character 14:1) supported the monophyly of the subfamily Saldinae. Chiloxanthinae with our fossil species included is a monophyletic group, which is supported by four synapomorphies: five well defined cells in membrane (Character 6:1); medial fracture long (Character 8:2); female subgenital plate truncate with concavity along the midline (Character 11:2); base of ovipositor exposed (Character 12:1). In summary, phylogenetic results suggest our new fossil genus is in Chiloxanthinae and short medial fracture was treated as a reversal character.

For the phylogenetic analysis including fossil species *Oligosaldina aquatilis*, we got one most parsimonious tree (Fig. 3D), tree length = 28, CI = 82, RI = 88. The monophyly of Saldidae is supported by four synapomorphies (Character 4:1, 9:1, 15:1 and 16:1) as the results above. In this phylogenetic result, besides Character 13:1 and Character 14:1, short costal fracture of hemelytra (Character 7:1) supports the monophyly of the subfamily Saldinae. Five well defined cells in membrane (Character 6:1) indicate that our new genus should be in the branch of Chiloxanthinae. Therefore, both of the phylogenetic analyses suggest our fossil species should be classified into Chiloxanthinae.

Comparison with Chiloxanthinae indicates the new fossil species differs from other extant chiloxanthines in its short medial fracture. Besides this character, the boundary between corium and membrane is not clear in *Enalosalda*, which is different from *Brevrimatus* gen. n. with clear boundary. *Paralosalda* has four membrane cells, which is different from *Brevrimatus* gen. n. with five cells. Embolar modification of female is well developed in *Pentacora*, but in *Brevrimatus* gen. n. the embolar region is slightly thickened. Sublateral cell of membrane is shortest in *Chiloxanthus*, which differs from *Brevrimatus* gen. n. with the lateralmost cell is shortest. We further compared it with other fossil Saldidae. The arrangement of the cells of *Brevrimatus* gen. n. is similar to that of *Oligosaldina*, but lateralmost cell of membrane is distinctly smaller than that of *Oligosaldina*. Long costal fracture is present on *Brevrimatus* gen. n., but absent on *Oligosaldina*. A deep furrow is present in the pronotum of *Oligosaldina*, while it is absent in our new genus*. Propentacora* contains five closed cells in the wing membrane, but the corial veinof *Propentacora* appears to continue between the third and fourth membrane cells, which is different from the new genus. *Brevrimatus* gen. n. is distinctly different from *Salda*, which can be seen in phylogenetic result. Comparing our fossils with the fossil species *Salda exigua*, we can separate them in the following characters: *Brevrimatus* gen. n. possesses five closed cells in the forewing membrane, while *Saldonia exigua* has three closed cells and rostrum of *Brevrimatus* gen. n. reaches to the base of hind coxae, while in *Saldonia exigua*, rostrum just reaches to the fore coxae. Therefore *Brevrimatus* gen. n. is different from all other fossil genera. In geological age, all of the previously recorded fossil saldids are from Cenozoic. So far, *Brevrimatus* gen. n. found in the Lower Cretaceous sedimentary stratum is the oldest saldid.

## Supplementary Material

XML Treatment for Brevrimatus

XML Treatment for Brevrimatus pulchalifer

## Figures and Tables

**Figure 4.**
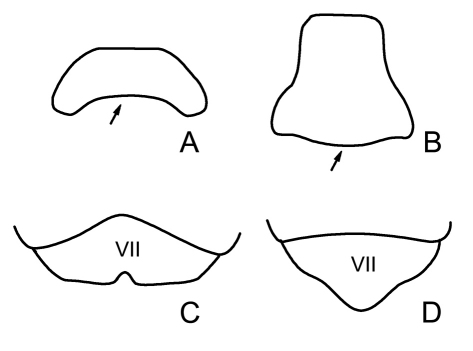
**A, B** pronotum **A** posterior pronotal margin indented **B** posterior pronotal margin non-indented **C, D** female subgenital plate **C** truncate with concave along the midline **D** posterior margin of produced caudad along the midline (modified from Polhemus and Chapman 1979).

## Appendix

### List of characters and character states used in phylogenetic analysis

**Distance between ocelli:** equal to width of ocellus (0); less than width of ocellus (1); more than width of ocellus (2). [We treat this character inapplicable in Aepophilidae that doesn’t have ocellus.]

**Rostrum reaches to:**

fore coxae (0); middle coxae (1); hind coxae (2). [Rostrum of Leptopodidae is very short, reaching to fore coxae at most. Rostrum of Saldidae is relatively long, reaching to middle coxae or hind coxae. Rostrum of *Brevrimatus pulchalifer* sp. n. and *Oligosaldina aquatilis* reaches to hind coxae. Long rostrum is the primitive character (Popov 1971).]

**Postclypeus:** absent (0); present (1). [Postclypeus present in *S. lugubris*, *T. altaica*, *S. montana*, *C. sichuanicus*. This character cannot be identified in our fossil specimen. In other groups postclypeus is absent.]

**Posterior pronotal margin:** non-indented (0); indented distinctly (1). [Posterior pronotal margin nearly straight in Aepophilidae, so we threat this character in Aepophilidae as non-indented. Posterior pronotal margin of Saldidae is demonstrated briefly in Figure 4A showing the indented posterior pronotal margin and Leptopodidae in Figure 4B showing the non-indented posterior pronotal margin. This character is considered as the synapomorphy for Saldidae.]

**Forewing:** macroptery or submacroptery (0); semibrachypterous moderately or strongly (1); brachyptery (2). [Wing polymorphism occurs in Saldidae. Wing pattern can be separated into five categories based on the reduction of the membrane of fore wing (Cobben 1980). The membrane of Aepophilidae completely reduced, so it is brachyptery. *C. sichuanicus* with the semibrachypterous forewing.]

**Cells:** Four well defined cells in membrane (0); five well defined cells in membrane (1). [In Aepophilidae, forewing greatly reduced, in form of pads without membrane, so we treated this character inapplicable in this family. Usually, Saldinae have four cells and Chiloxanthinae have five cells, but the distinction in hemelytral cells between the Saldinae and Chiloxanthinae is not constant (Polhemus and Chapman 1979). The fossil species, *B. pulchalifer* sp. n. and *O. aquatilis* have five cells.]

**Costal fracture of hemelytra:** absent (0); present, short (1); present, very long (2). [Costal fracture length is a stable character within subfamily. Saldinae usually possesses short costal fracture and Chiloxanthinae usually possesses long costal fracture. Polhemus (1977) consider the long costal fracture in Chiloxanthinae to be a derived character providing a synapomorphy for the group. Costal fracture is absent in *P. thaiensis*, *A. bonnairei* and *O. aquatilis*. Costal fracture in our fossil species, *B. pulchalifer* sp. n., is long.]

**Medial fracture:** absent (0); present, short (1); present, very long (2). [Long medial fracture reaches at least to level of posterior end of claval suture, and short medial fracture not reaches anteriorly more than half the distance from costal fracture to posterior end of claval suture (Schuh and Slater 1995). Medial fracture is absent in *A. bonnairei*. In *P. thaiensis*, *B. pulchalifer* sp. n. and members of Saldinae, medial fracture is short. *C. pilosus*, *P. ligata* and *P. innova* have long medial fracture.]

**Eversible glands:** absent (0); present posterolaterally between sterna VI and VII (1). [Saldidae has paired abdominal eversible glands with their openings locating between sterna VI and VII. Eversible glands is absent in Leptopodidae and Aepophilidae. This structure is unknown in *B. pulchalifer* sp. n. and *O. aquatilis*. Eversible glands is considered to be a apomorphic character in Saldidae (Polhemus 1977).]

**S****clerite adjacent to eversible gland:** present (0); absent (1). [This sclerite is present in Saldinae, but absent in Chiloxanthinae.]

**Female subgenital plate:** truncate without concavity along the midline (0); triangular, posterior margin of produced caudad along the midline (1); truncate with concave along the midline (2). [In *A. bonnairei* female subgenital plate just like a normal abdominal sternite. Subgenital plate is truncate with concave along the midline is found in the members of Chiloxanthinae and *B. pulchalifer* sp. n. as Figure 4C. Subgenital plate is triangular in Saldinae as Figure 4D]

**Base of ovipositor:** hidden by posterior medial prolongation of subgenital plate (0); exposed (1). [The base of ovipositor is hidden in Aepophilidae and Saldinae, but exposed in Chiloxanthinae and *B. pulchalifer* sp. n. The exposed ovipositor is considered a derived character (Polhemus 1977)]

**Apicolateral sclerotized structures of penis:** absent (0), present (1). [This structure absent in Aepophilidae and Chiloxanthinae, but present in Saldinae.]

**Filum gonopori:** base of penis-filum not curled or at most forming one closed ring (0); filum gonopori coiled one to four times, like a watch-spring (1). [Base of penis-filum not curled in Leptopodidae and Aepophilidae, and coiled less than one ring in Chiloxanthinae. In Saldinae, base of penis-filum like a watch-spring.]

**Eggs:** without aeropyles (0); with aeropyles (1). [Eggs with aeropyles is regarded as a synapomorphy for Saldidae.]

**Larval organ:** absent (0); present (1). [Larval organ is absent in Leptopodidae and Aepophilidae, *S. lugubris* and *T. altaica*. Larval organ present in *S. montana*, *C. sichuanicus*, *C. pilosus*, *P. ligata*, *P. innova*. Larval organ present in most members of Saldidae. The absence condition in Saldini is presumed to be the secondary lost (Polhemus 1977).]

**Larval organ:** larval organ lateral, adjacent to spiracle (0); larval organ located medially, some distance from the spiracle (1). [In Saldinae, larval organ when present adjacent to spiracle, while the later condition occurred in Chiloxanthinae.]
